# Association of claw disorders with claw horn colour in norwegian red cattle - a cross-sectional study of 2607 cows from 112 herds

**DOI:** 10.1186/1751-0147-53-59

**Published:** 2011-11-15

**Authors:** Åse M Sogstad, Terje Fjeldaas, Olav Østerås

**Affiliations:** 1 Norwegian Cattle Health Services, TINE Extension Services, Pb 58, 1431 Ås, Norway; 2 Norwegian School of Veterinary Science, Pb 8146, Dep., 0033 Oslo, Norway

## Abstract

Claw disorders cause problems in dairy cattle all over the world. Nutrition, feeding, environment, claw trimming routines, hormonal changes related to calving and genetics are among the factors which influence the pathogenesis. The colour of the claw horn (pigmentation) has been suggested to play a role. The aim of this study was to investigate if there were any associations between the colour of the sole horn and claw disorders detected at claw trimming. Altogether, 2607 cows on 112 farms were claw trimmed once and the colour (dark, mixed or light) of the right lateral hind claw and hind claw disorders were recorded by 13 trained claw trimmers. The data were analysed using logistic regression models with logit link function, binomial distribution and herd and claw trimmer as repeated effects, with herd nested within claw trimmer. Haemorrhages of the sole (HS) and white line (HWL) were more frequently found in light than in dark claws (OR = 2.61 and 2.34, respectively). Both HS (OR = 1.43) and corkscrewed claws (OR = 1.84) were slightly more prevalent among cows which had claws with mixed colour versus dark claws. There were no significant associations of other claw disorders with claw horn colour.

## Findings

Claw disorders in cattle cause poor animal welfare and economic losses around the world. The pathogenesis is mainly influenced by nutrition, feeding, environment, claw trimming routines, hormonal changes related to calving and genetics (breed, claw shape etc) [1] as well as horn quality [2]. Many of the mentioned factors are important for the pathogenesis by affecting horn quality [3-6]. Also, the claw diseases themselves influence the production of horn and thereby the quality. Good quality horn is more able to withstand the mechanical forces of hard, uneven floors and slurry in the environment. Furthermore, the characteristics of the horn are dependent on the number of horn tubules per unit area and the water, mineral and protein content [7]. Hard horn might be more able to resist mechanical forces and moisture and hence be more resistant to claw disorders [7,8]. It has been proposed that dark-coloured (pigmented) horn is more resistant to mechanical forces and moisture than light-coloured (non-pigmented) horn [9], although Chesterton et al. [10] found that the influence of colour on lameness was highly influenced by breed. A review by Vermunt and Greenough [2] revealed that the results from various studies were not consistent.

This study was part of a project on lameness and claw disorders in Norwegian Red dairy cattle [11] and our aim was to investigate whether there were any associations between claw disorders revealed at claw trimming and the colour of sole horn.

The final study population consisted of 2607 cows from 55 tie stalls and 57 free stalls. Thirteen professional claw trimmers were specifically trained by the authors in a course immediately before the claw trimming visits, as well as by individual training for each trimmer in one or two herds at the start of the project. The trimmers performed one visit per farm with claw trimming and recording of hind claw disorders (Table 1). Only the colour of the sole of the right lateral hind claw was recorded because the wall of most cows were covered by manure and also because we had to make the procedures as practical as possible for the claw trimmers. The sole was recorded as being predominantly dark - coloured, mix - coloured or light - coloured. In mix-coloured claws approximately half of the sole was dark and the other half light. More information about the selection procedure, the study population, the training of claw trimmers and the recording of disorders is in [11].

**Table 1:** Definition claw lesions recorded at trimming

Lesion	Score	Definition
Dermatitis	1	Superficial, hyperaemic, slightly exudative lesion of the digital/interdigital skin
	2	Exudative, slightly ulcerative lesion with thickening of the skin
	3	Ulcerative, spontaneously bleeding lesion with thickening of the skin and great pain
Heel-horn erosion	1	Slight defects of the horn integrity, pits and small fissures
	2	V-shaped fissures or craters of the heel/bulb not affecting corium
	3	V-shaped profound fissures or craters affecting corium of the heel/bulb
Haemorrhages of the white line	1	Slight haemorrhagic discoloration
	2	Moderate haemorrhage on a single spot or several superficial haemorrhages covering > 20% of the white line
	3	Profound haemorrhage on a single spot or extensive haemorrhagic discoloration covering > 50% of the white line
Haemorrhages of the sole	1	Slight haemorrhagic discoloration
	2	Moderate haemorrhage on a single spot or several superficial haemorrhages covering > 20% of the sole surface
	3	Profound haemorrhage on a single spot or extensive haemorrhagic discoloration covering > 50% of the sole
Sole ulcer	1	Exposed, unaffected corium
	2	Granulation tissue, necrosis, purulent exudates and separation of the sole horn
	3	As score 2 with additional affection of the deeper structures of the claw
White-line fissure Corkscrewed claws	1	Fissure, which disappear with deep cut beneath normal trimming level
	2	Deep fissure perforating next to the corium of sole or wall
	3	Corium is affected with purulent exudates, eventually with necrosis, granulation tissue and separation of the wall and/or sole Included both mild cases, in which the abaxial wall was bent inwards, with a curved dorsal border, and serious cases of corkscrew claws, in which the abaxial wall was part of the weight-bearing surface.
		
		

Data on herd and individual characteristics were collected from the Norwegian Cattle Health Recording System. The analyses were performed at cow level. Alternating logistic regression models [12] were fit using "Proc Genmod" with logit link function, binomial distribution and herd and claw trimmer as repeated effects, with herd nested within claw trimmer: Logit(p_i_) = β_0 _+ β_1_X_1i _+......+ β_k_X_ki _+ Z'_iht _where × represent the fixed effect and Z'_iht _the *z*-matrix for each cluster *i *and each unique within-cluster pair (herd (*h*), and clawtrimmer (*t*). The claw disorders were initially scored as mild, moderate or severe, but few cows had severe lesions and no significant associations were found when moderate and severe scores were analyzed separately. Consequently, all scores were added together and the outcome was binomial. The outcome variables were dermatitis (D), heel-horn erosion (HHE), haemorrhage of the sole (HS), haemorrhage of the white line (HWL), sole ulcer (SU), white-line fissure (WLF) and corkscrewed claw (CORK). Fixed effects included in the model were type of stall (free or tie), parity and months in milk (MIM). These were chosen on the basis of experience from previous studies [13]. The final model was produced by stepwise backward elimination. The variable with the highest *P*-value was excluded until all variables in the model had a *P*-value of £0.05.

Haemorrhages of the sole and white line were more frequently found in light than in dark claws (OR = 2.61 and 2.34, respectively) (Table 2). Both HS (OR = 1.43) and CORK (OR = 1.84) were slightly more prevalent in mix-coloured versus dark claws. There were no significant differences for other claw disorders. There were fewer HS, HWL and CORK in tie stalls than in free stalls (OR = 0.51, 0.49 and 0.51, respectively). There were more HS in cows in the first lactation than in older cows (OR = 1.43), and there were fewer CORK in cows in the first lactation than in older cows (OR = 0.63)(Table 2). The effect of MIM is indicated in a footnote in Table 2. Haemorrhages of the sole and the white line were most prevalent approximately 4-6 months after calving and CORK showed a slow increase with MIM.

**Table 2:** Odds ratio (95% CI) for the significant (*P *< 0.05) associations between claw horn colour (light, mixed or dark) and the following claw disorders haemorrhages of the sole (HS), haemorrhages of the white line (HWL) and corkscrewed claws (CORK) in 2607 hind claws, adjusted for stall type (tie or free), parity and month in milk (MIM)^4 ^and the random effects of herd and claw trimmer

Variable	Class	n	HS^1^	HWL^2^	CORK^3^
Colour	Light	1163	2.61 (2.23/3.05)	2.34 (1.96/2.79)	1.54 (0.92/2.56)
	Mixed	153	1.43 (1.11/1.85)	1.46 (1.00/2.12)	1.84 (1.15/2.95)
	Dark	1291	1.00	1.00	1.00
Stall	Tie	1091	0.51 (0.39/0.68)	0.49 (0.36/0.67)	0.51 (0.29/0.89)
	Free	1516	1.00	1.00	1.00
Parity	1	1021	1.43 (1.16/1.78)		0.63 (0.48/0.82)
	2	708	1.02 (0.82/1.27)		1.06 (0.68/1.67)
	≥ 3	878	1.00		1.00
Random effects					
Herd			1.80 (1.27/2.57)	2.03 (1.49/2.78)	1.73 (1.15/2.62)
Claw-trimmer			1.48 (1.08/2.02)	1.32 (1.01/1.74)	1.00 (0.89/1.12)

^1^Numbers of cows with light-, mixed- and dark-coloured claws and HS were 268, 26 and 136, respectively

^2 ^Numbers of cows with light-, mixed- and dark-coloured claws and HWL were 176, 15 and 93, respectively

^3 ^Numbers of cows with light-, mixed- and dark-coloured claws and CORK were 59, 10 and 43, respectively.

^4^Estimates (SE) for MIM and MIM*MIM were 0.48 (0.10) and -0.05 (0.01) for HS, 0.26 (0.05) and -0.03 (0.01) for HWL and 0.05 (0.02) for CORK (only MIM).

We performed the analyses on cow level. This has hopefully not influenced the results significantly. Personal experience tells us that Norwegian Red cows usually are symmetrical coloured with the same colour of the coronary band on both hind limbs. Consequently, it is most likely that the colour of the two hind claws within one cow was approximately the same.

We did not measure horn hardness in the present study. According to Feder [14] and McDaniel [15], dark-coloured horn is about 30% harder than light horn. In contrast, Hepburn et al. [16] discovered that light-coloured wall horn demonstrated significantly greater values for hardness than dark horn. However, they found no differences between dark and light horn either in the sole or the heel. This is partly in agreement with Clark and Rakes [17], who reported that horn hardness was not related to horn colour. Horn hardness is affected by individual factors, but is also highly influenced by the amount of moisture and manure in the environment [6,7]. O'Driscoll et al. [18] on their side, found a low correlation between horn hardness and claw pathologies in a study on the effect of out-wintering pad design.

Few studies have investigated the direct relationship between colour of claw horn and different claw disorders. We found association between the laminitis - related lesions HS and HWL and light claws. Chesterton et al. [10] found light claws of Friesian cows to be more prone to lameness than the darker claws of Jersey cows. This partly agrees with our results, because HS and HWL can develop into SU and WLF, which cause lameness [11].

When interpreting the results, it should be kept in mind that it is possible that haemorrhages are more easily spotted in light than in dark claws, and this might have influenced our results.

There were no significant associations between the most lameness-causing claw disorders and colour of the sole horn in our study.

We have no explanation for why CORK was associated with a mixed colour in our study other than there might be some correlated genetic traits involved.

The random effect of herd being significant for all claw disorders, suggests that the herd- specific factors management including claw trimming, feeding and cleaning routines, housing and nutrition are important. The effect was least marked for CORK, suggesting that individual-cow factors are more important than herd factors for this disorder. The effect of claw trimmer was low for most lesions and very low for CORK, indicating that differences in recording of claw colour and diseases between claw trimmers had minor effect on the outcome.

## Conclusion

Because colour was not associated with most claw disorders, our study allows for the conclusion that susceptibility to claw disorders is probably more affected by factors other than claw horn colour (pigmentation). However, the associations found between colour and haemorrhages indicate that the colour might be of some importance and that controlled studies, including measuring horn hardness, are needed to decide whether claw horn colour should be included in breeding programs.

## Competing interests

The authors declare that they have no competing interests.

## Authors' contributions

ÅMS worked together with TF in the planning stage of the study and with the collection of data and is the first author of the paper. TF has also contributed to the paper writing. OØ contributed in the planning stage of the study and with statistical help. All authors read and approved of the final manuscript.

## Acknowledgements

The authors gratefully acknowledge the participating claw trimmers and farmers. They also acknowledge Kerstin Plym Forshell, Norwegian Cattle Health Services for her contributions in the initiation phase of the project. The study was funded by TINE Norwegian Dairies, GENO Breeding and A.I. Association, Animalia and The Research Council of Norway. Access to data was given by the Norwegian Dairy Herd Recording System and the Norwegian Cattle Health Services in agreement number 6/2001 by 19.09.2001.
